# MOBAK 3–4: Construct Validity and Score Reliability in an 8–10-Year-Old Portuguese Sample Within the Cascais Municipality

**DOI:** 10.1177/00315125251401274

**Published:** 2025-12-18

**Authors:** João Mota, Afonso Meira, João Martins, Marcos Onofre, Maria João Martins

**Affiliations:** 1 School of Education, Physical Education, Sports Studies and Arts Programme, 8795 University College Cork, Cork, Ireland; 2 Centro de Estudos em Educação, Faculdade de Motricidade Humana, 37809 Universidade de Lisboa, Lisboa, Portugal; 3 UIDEF, Instituto de Educação, 37809 Universidade de Lisboa, Lisboa, Portugal

**Keywords:** MOBAK 3-4, physical education, factor analysis, assessment, motor competence, validity

## Abstract

**Background:** Developing children’s motor competence (MC) is central to fostering physical literacy and constitutes a core aim of high-quality physical education. Accurate and valid assessment tools are therefore essential. The MOBAK 3–4, following the MOBAK 1–2, was designed to assess basic motor competencies (BMC) in 8–10-year-olds. **Purpose:** This study aimed to provide evidence of construct validity and score reliability for the MOBAK 3–4 in a Portuguese sample. **Study Sample: **A total of 436 pupils (M = 9.4 ± 0.6 years; 53% boys) were assessed by trained test administrators with excellent inter- and intra-rater agreement. **Results: **Confirmatory factor analysis supported a two-factor correlated model—Object Movement (OM) and Self-Movement (SM)—including residual covariances between Dribbling–Running and Balancing–Jumping. Stepwise measurement invariance testing across sex supported partial thresholds and loadings invariance (Throwing and Running freed). Latent mean comparison indicated boys scored significantly higher in OM (d = 0.87 [0.86, 1.63]), but similarly in SM (d = −0.29 [−0.57, 0.06]) A Multiple Indicators Multiple Causes model with age evidenced the moderating effect of sex: age predicted higher OM and SM in girls, but negligible gains in boys. Score reliability was acceptable for OM (Ω = .69) but inadequate for SM (Ω = .39), limiting its interpretability as a stand-alone scale, particularly in girls. Regression-based OM and SM subscores are recommended over a single global index. **Conclusions:** MOBAK 3–4 is a feasible and psychometrically supported tool for assessing children’s BMC. Results highlight age- and sex-specific patterns in MC, with implications for research, policy, and practice in physical education.

## Introduction

Over the past decade, research has consistently shown a strong link between motor competence (MC), physical activity, and health-related fitness in children and adolescents (Barnett et al., 2021; Cattuzzo et al., 2016; Cohen et al., 2015; Holfelder & Schott, 2014; Logan et al., 2015; Lubans et al., 2010; Robinson et al., 2015; Stodden et al., 2008). As such, it can be regarded as a crucial component within the physical domain of physical literacy—a set of holistic skills, attitudes and knowledge that underpin lifelong and significant participation in physical activity (Sport Australia, 2019; Whitehead, 2019)—a key goal of any high-quality physical education (PE) curriculum (United Nations Educational, Scientific and Cultural Organization [UNESCO], 2015). The first step in the process of developing MC is teaching children fundamental movement skills (FMS) during the early school years.

FMS are basic movement patterns that form the basis for more complex, specialized skills required for successful participation in recreational, competitive, and daily living physical activity (Goodway et al., 2020). These basic, observable patterns of behavior—which include locomotor (e.g., running, jumping, leaping, sliding, galloping, skipping, hopping), manipulative (e.g., throwing, catching, dribbling, striking, volleying, kicking) and stability (e.g., balancing on one foot, walking on a beam, axial movements) skills—progress through a defined developmental process from immaturity to proficiency, influenced by task-specific, individual, and environmental factors. Since FMS development is not solely dependent on maturation, children aged 6 to 10 require the right conditions to achieve proficiency. These include opportunities for deliberate and structured practice, feedback, and developmentally appropriate instruction (Barnett, Stodden, et al., 2016; Goodway et al., 2020; O’Brien et al., 2016); all more readily found in PE settings rather than free-play environments.

Assessment, both for formative and summative purposes, can play an essential role in children’s MC development in PE, particularly with the growing emphasis on inclusion in education. It can provide teachers with data about their students’ learning needs and outcomes. In fact, educational testing has gained importance in PE, with several tools being designed for diagnosis and/or monitoring of children’s MC (Scheuer, Herrmann, & Bund, 2019). One such tool is MOBAK (German: *Motorische Basiskompetenzen*), a test battery that is adjustable to different grades, including versions such as the MOBAK 1–2 (Herrmann et al., 2015), for the 1st and 2nd grades, and the MOBAK 3–4 (Herrmann & Seelig, 2017), for the 3rd and 4th grades. This assessment tool provides data on children’s basic motor competencies (BMC), a term that is associated with the aforementioned FMS but that, instead of movement-specific and process-oriented (i.e., focused on the critical elements of motor skills), is context-specific and product-oriented (i.e., focused on the completion of tasks with motor skills).

Evidence supporting the validity of MOBAK 1–2 to assess Portuguese children has been previously published (Quitério et al., 2018). However, such evidence for MOBAK 3–4 is still lacking, despite being previously used in a study by Carvalho et al. (2024). Addressing this gap is critical, as it would allow Portuguese PE teachers to assess MC across primary school grades using a consistent test battery. Additionally, it would enable researchers in motor control, learning, and development to explore sociodemographic differences in MC, contributing to a deeper understanding of motor skill acquisition in Portuguese children.

Given the importance of assessing primary school children’s MC in PE to foster physical literacy and inform research-driven policy and practice, this study aims to determine the construct validity and score reliability of the MOBAK 3–4 test instrument.

## Methods

### Participants

A total of 436 pupils (M = 9.4 ± 0.6 years; 53% boys), 220 in 3rd grade and 216 in 4th grade, were recruited from 22 primary schools in Cascais (Lisbon, Portugal), representing all 11 school clusters in the municipality. Ethical approval was granted by the Ethical Committee of the Faculty of Human Kinetics – University of Lisbon (CEIFMH N°: 02/2023) as part of the project *Avaliação da competência motora das crianças do 1.° ciclo do ensino básico de Cascais* (co-funded by the Cascais Municipal Council). Written consent was obtained from legal guardians, assent from children, and approval from school principals prior to data collection.

### Measures/Instruments

Motor competence was assessed using the MOBAK 3–4 (Herrmann & Seelig, 2017), which includes eight items grouped into two domains of basic motor competencies (BMC): (A1) Object Movement (OM)—Throwing, Catching, Bouncing, Dribbling; and (A2) Self-Movement (SM)—Balancing, Rolling, Jumping, Running. Each item is scored 0–2 points, yielding a maximum of 8 per domain. For Throwing and Catching, pupils had six attempts (0 = 0–2 successes; 1 = 3–4; 2 = 5–6). For the remaining six items, pupils had two attempts (0 = none successful; 1 = one successful; 2 = both successful).

### Data Collection

Secondary school students enrolled in PE/sport vocational courses (three clusters) were recruited and trained as test administrators. Training consisted of four days (January 2024) combining peer-assessment exercises and protocol routines, followed by two rounds of video-based scoring, spaced over a week. Inter- and intra-observer reliability, assessed via Bellack’s index (Bellack, 1968), was excellent (=.98; Nunnally & Bernstein, 1994; Price, 2017). Students meeting the ≥ .80 cut-off served as test administrators (*n* = 24); the remainder acted as group guides (*n* = 24).

After a pilot session with live assessments, main data collection occurred over 11 days (February–March 2024) in sports halls across Cascais. Each session (∼90 min) tested one 3rd- and one 4th-grade class simultaneously. Pupils rotated through eight stations, each supervised by a trained administrator who provided standard instructions and a single demonstration per item.

## Data Analysis

All subsequent statistical analyses used RStudio 2025.05.1 + 513 (Posit team, 2025), with *R* 4.5.0 (R Core Team, 2025). Descriptive statistics were computed using the *gtsummary* package (Sjoberg et al., 2021). No missing data were observed in the dataset. Bivariate Spearman correlations between variables were obtained in *rstatix* (Kassambara, 2021).

### Confirmatory Factor Analysis

Previous literature has proposed an *a priori* factorial structure for MOBAK 3–4 (Herrmann & Seelig, 2017). To test this hypothesized model structure, as well as several competing models, we employed Confirmatory Factor Analysis (CFA). We estimated four models: one unidimensional model and three variations of a two-factor correlated model. Models were estimated in *lavaan* 0.6.19 package (Roseel, 2012), using robust means and variance-adjusted weighted least squares estimator (WLSMV) under delta parameterization. Latent variance was fixed to one in all models (std.lv = TRUE). Residual covariances were set to zero unless otherwise specified. All models converged satisfactorily.

#### Model Fit and Selection

All indices used to assess model fit are summarized in Table 1 along with their purpose and guidelines. The different thresholds for SRMR, CFI, and RMSEA were used holistically to analyze global fit, rather than as strict cut-offs (Chen et al., 2008; Gana & Broc, 2019; Hu & Bentler, 1999; Marsh et al., 2004; Schreiber et al., 2006). Nested models were compared using the Satorra-Bentler robust χ^2^ difference test; non-nested models were compared resorting to the assessment of all available indices and tests.

**Table 1.** Summary Table of Guidelines Used for Assessing Construct Validity and Score Reliability

Index/Statistic	Purpose	Guideline	Descriptor	Reference
Scaled χ^2^	Absolute Fit	*p* > .05		(Hu & Bentler, 1999; Schreiber et al., 2006)
SRMR		<.08		
Robust RMSEA and RMSEA	Approximate Fit	≤.06		
Robust RMSEA 90% CI and RMSEA 90% CI		.10 not included in interval		
Robust CFI and CFI		≥.95		
Modification Indices	Local fit	<3.84		(Brown, 2015)
Absolute Correlation Residuals		<|.10|		(Kline, 2023; Maydeu-Olivares, 2017)
Standardized Factor Loading	Convergent Validity	>.71	Excellent	(Comrey & Lee, 1992)
		>.63	Very Good	
		>.55	Good	
		>.45	Fair	
		>.32	Poor	
Inter-factor correlations	Discriminant Validity	<.85		(Brown, 2015)
Omega coefficient (ω)	Total score reliability	>.80	Good	(Price, 2017)
		>.70	Acceptable	(Nunnally & Bernstein, 1994)

*Note.* CFI = Comparative Fit Index; RMSEA = Root Mean Square Error of Approximation; SRMR = Standardized Root Mean Square Residual; CI = Confidence Interval.

#### Measurement Invariance and Latent Means

Measurement invariance (MI) across sex was evaluated using Multiple-Group CFA, following recommended stepwise procedures for ordinal indicators (Millsap & Yun-Tein, 2004; Svetina et al., 2020; Wu & Estabrook, 2016). First, thresholds-only invariance was tested (equating thresholds across groups), followed by thresholds + loadings (equating thresholds and loadings across groups). When full invariance was untenable, partial invariance was achieved by freeing the factor loadings of Running and Throwing, guided by modification indices and expected parameter change values. Evaluation of the solutions used a combined threshold of ΔCFI ≤ .010 and ΔRMSEA ≤ .015 (Chen, 2007; Cheung & Rensvold, 2002), alongside the robust χ^2^ difference test. Latent mean comparisons were interpreted under the partial invariance solution.

#### Score Reliability and Known-Groups Validity

Total score reliability (for the unidimensional model) and dimensional score reliability were assessed using the omega coefficient (Raykov, 2001) within the *semTools* package (Jorgensen et al., 2021). A multiple indicator, multiple causes model (MIMIC) model was run using the partial invariant solution and including age as a covariate to assess known-groups validity and test sex moderation effects. For the moderation test, structural regressions from age to both latent factors were estimated under equality-constrained and freely estimated models. Grand-centering of age was required to achieve convergence in the equality-constrained model; therefore, the same procedure was applied to the freely estimated model to ensure comparability; results are otherwise reported from the non-centered solution. The same estimation procedures and assessment criteria described for CFA and MI were applied.

## Results

### Inter and Intra-Observer Reliability

Out of 48 prospective test administrators, 24 achieved a good intra and inter-observer index (Bellack’s) of .80 and were thus selected. The intra and inter-observer indexes were both .98 when considering the final set of administrators.

### Item Score Distribution

**Figure 1.**
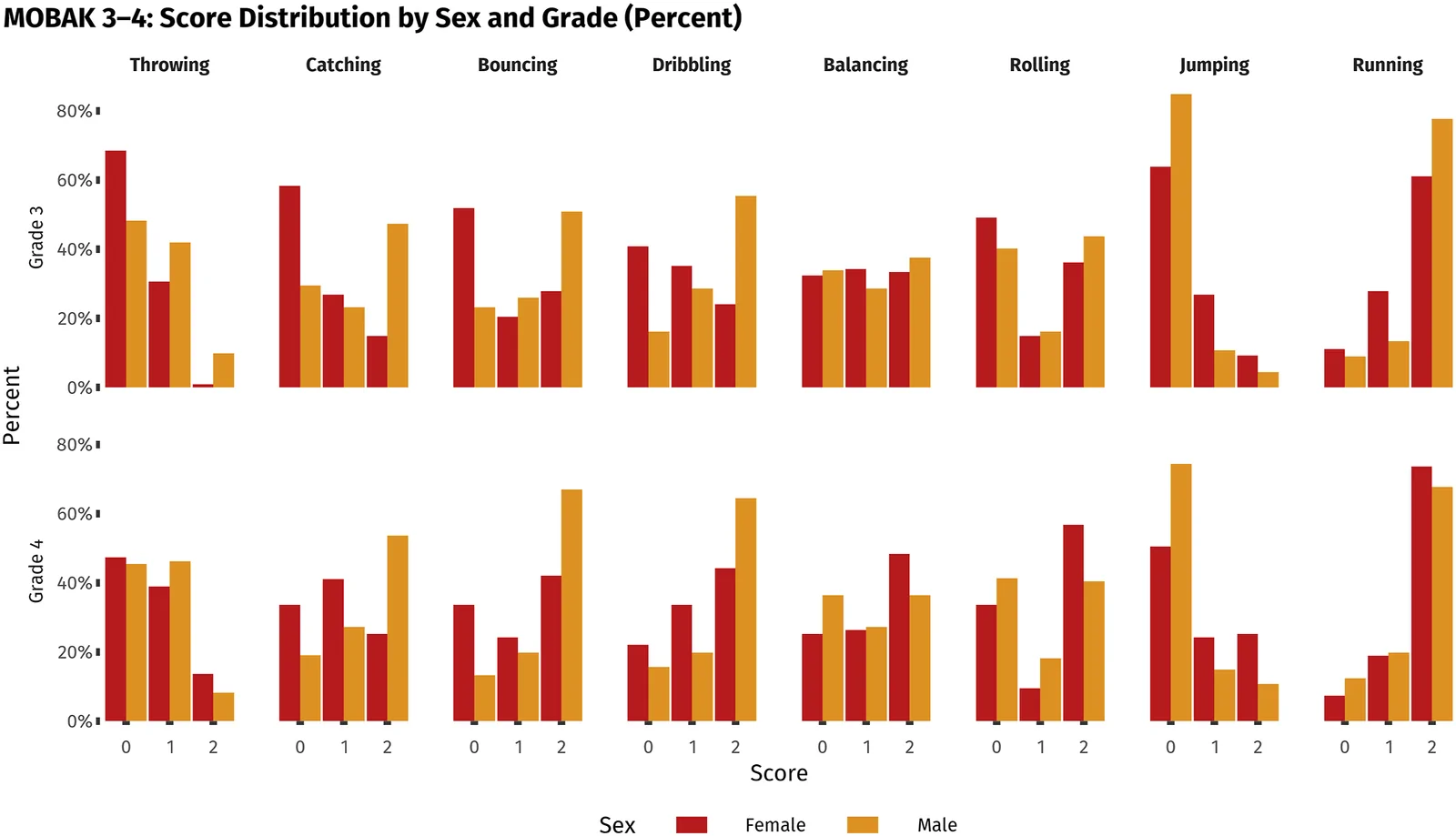
Score Distribution by Sex and Grade

Considering the number of students obtaining 0 points, Jumping seemed to be the hardest test for both girls and boys, with boys scoring lower than girls, while Running seemed to be the easiest. This general pattern was maintained across grades. Girls tended to score lower scores in Throwing, Catching, Bouncing and Dribbling than boys (Figure 1, full percentages available in Supplemental Table 1 in Supplemental File 1), but this difference was attenuated by grade.

### Correlations

The bivariate correlations between all items for students of different sexes and ages (Table 2) reflect the earlier descriptive results regarding score distribution based on sex and grade. Older students tend to achieve slightly higher scores in Throwing, Catching, Bouncing, Dribbling, and Jumping, as indicated by the small to medium (Gignac & Szodorai, 2016), but statistically significant, estimates.

**Table 2.** Bivariate Spearman Correlation Coefficients

Item	1	2	3	4	5	6	7	8
1. Throwing	—							
2. Catching	.**24**	—						
3. Bouncing	.**22**	.**44**	—					
4. Dribbling	.**11**	.**38**	.**46**	—				
5. Balancing	**.**09	**.11**	.**10**	**.11**	—			
6. Rolling	.06	.**22**	.**18**	.**19**	.**21**	—		
7. Jumping	.**10**	.05	.**16**	.**13**	.**24**	.**20**	—	
8. Running	.09	.**21**	.**15**	.**23**	.06	.**14**	.02	—
								
Sex	.**12**	.**32**	.**29**	.**27**	−.06	−.01	−.**23**	.05
Age	**.15**	**.13**	**.23**	**.16**	.03	.08	.**12**	−.04

*Note.* Female used as the reference group for sex correlation; statistically significant (*p* < .05) estimates are bolded.

Tests for OM (Catching, Bouncing, Dribbling) showed moderate to strong statistically significant correlations with each other, stronger than with tests for SM (Balancing, Rolling, Jumping, Running). The Throwing test, also part of OM, had a less salient correlation pattern within this dimension. SM tests had small to medium correlations with each other, except for the Running test, which did not correlate with other SM tests but had small to medium statistically significant correlations with OM tests.

### Confirmatory Factor Analysis

#### Model Fit

The unidimensional model (M1) fitted poorly to our data (see Table 3), failing both the exact fit test and attaining mostly poor indices of approximate fit. This provides evidence against the use of a sum score for the total MOBAK 3–4 score that encompasses all eight tests.

**Table 3.** Summary of Model Fit, Including Global Fit, Approximate Fit, Factor Correlations, Standardized Loadings, and Score Reliabilities

Fit measure	Unidimensional (M1)	Correlated factors (M2a)	Correlated factors (M2b)	Correlated factors (M2c)
WLSMVχ^2^	66.090 (20), *p* < .001	39.843 (19), *p* = .003	29.739 (18), *p* = .040	24.781 (17), *p* = .100
Robust CFI	.86	.93	.95	.96
Robust RMSEA [90% CI]	.10 [.07, .14]	.08 [.04, .11]	.06 [.02, .10]	.06 [.00, .10]
SRMR	.07	.06	.05	.05
Factor Correlations (SE)				
Object Control ∼ Locomotion		.61 (.07)	.66 (.07)	.61 (.08)
Score Reliability (Omega)	.66			
Object Movement		.69	.69	.69
Self-Movement		.44	.38	.39
Std. Loadings	.29 – .77			
Object Movement		.37 – .80	.36 – .79	.37 – .80
Self-Movement		.41 – .59	.34 – .59	. 35 −.62

*Note.* CFI = Comparative Fit Index; RMSEA = Root Mean Square Error of Approximation; WLSMV = weighted least square mean and variance adjusted; SRMR = Standardized Root Mean Square Residual; SE = standard error;

M2a = baseline 2-correlated factor MOBAK model; M2b = Residual correlation freed between Balancing and Jumping tests; M2c = Residual correlation freed between Balancing and Jumping tests, and Dribbling and Running tests.

The two-correlated factors model (Model 2a) fitted the data better, as per a statistically significant WLSMV χ^2^ robust difference test, (1) = 17.78, *p* < .001. Although global fit indices were close to conventional cut-offs, examination of modification indices (MI) revealed local misfit; most prominently for residual covariances involving Running, which showed cross-factor overlap with items from both OM and Self Movement. Specifically, the largest MI (12.18) suggested a cross-loading of Running on OM, while additional high MIs were observed for residual associations between Balancing–Jumping (MI = 8.01), Jumping–Running (5.29), and Dribbling–Running (4.82). Smaller, yet non-trivial MIs were present for Catching–Jumping (4.46), Catching–Running (3.86), and Throwing–Dribbling (3.38). To assess whether freeing these dependencies improved model fit without altering the intended factor structure and content validity, two further, theoretically plausible, respecified models were tested.

Given the size of misfit and theoretical plausibility — both tasks emphasize dynamic postural control and balance recovery under load — the Balancing–Jumping residual covariance was freed first. This respecification yielded statistically significant improvement, Δχ^2^(1) = 8.807, *p* = .003, and a borderline exact global fit, with all fit indices approaching recommended thresholds. Analysis of local fit, revealed that albeit reduced, there was still a significant suggested cross-loading of Running on OM (MI = 8.24), followed by a non-plausible cross-loading of Rolling on OM (4.63). In an attempt to strike a balance between content validity and fit, we freed the next plausible residual covariance (Dribbling-Running, 3.36) to model some of the shared variance between Running and the OM factor, resulting in Model 2c.

Model 2c had a significant improvement upon Model 2b, Δχ^2^(1) = 5.278, *p* = .022, considering both the χ^2^ test and relative fit indices. Local fit results still flagged the association of Running on OM, but to a smaller degree (5.75; full residual matrix available in Supplemental Table 2). Given that releasing further covariances would likely result in overfitting to our data, we retained Model 2c as the best theoretical-empirical fit.

#### Convergent and Discriminant Validity, Score Reliability

**Figure 2.**
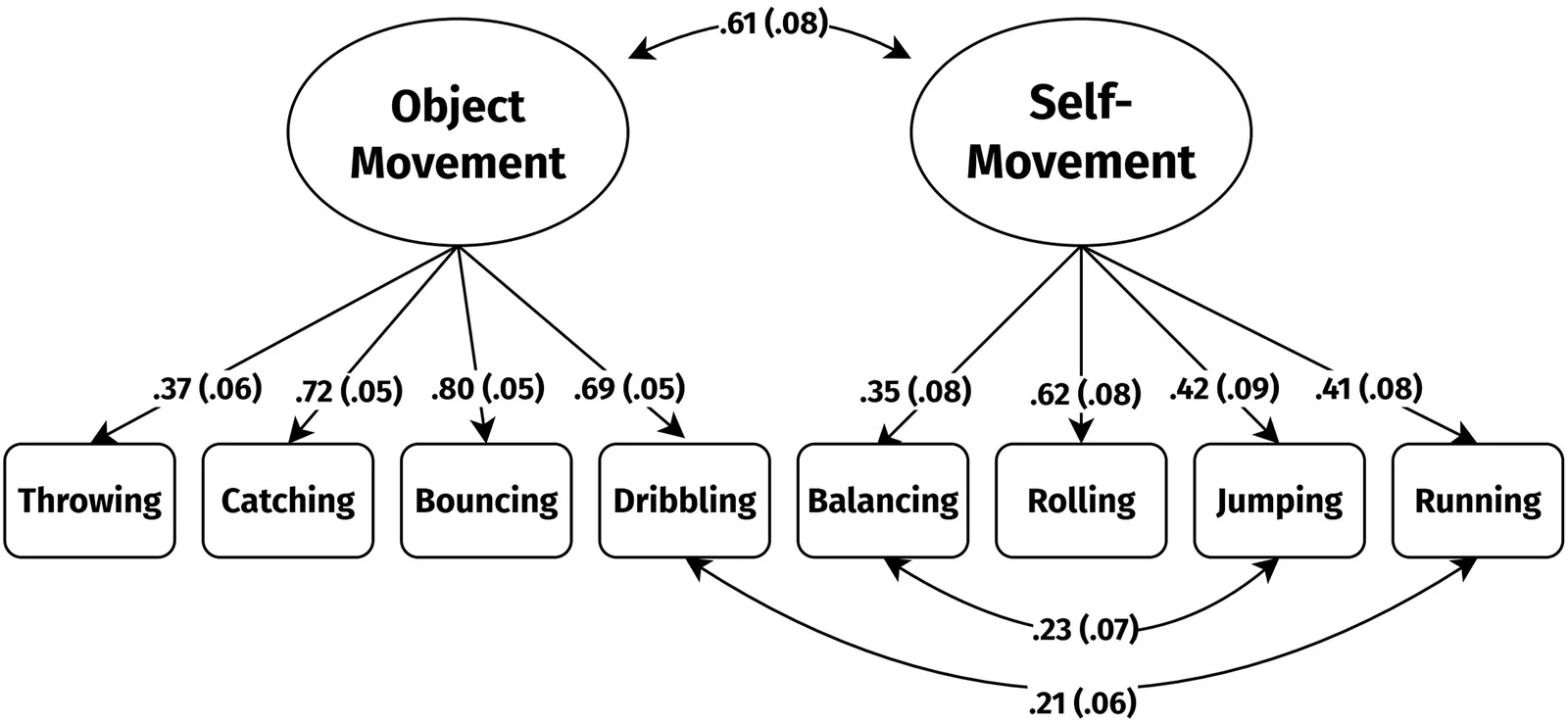
Confirmatory Factor Analysis results (Model 2cd), Fully Standardized Solution. All Standardized Loadings, and Factor and Residual Covariances Were Statistically Significant

Standardized loadings in Model 2c were mostly very good and excellent for the OM factor, aside from the Throwing test, supporting its overall convergent validity (Figure 2). However, loadings for tests in SM factor were lower with mostly poor-fair loadings, excluding Rolling which attained a good loading; highlighting that this factor may not account for a significant amount of the variance of its tests and thus attaining insufficient evidence for its convergent validity. Nonetheless, correlation between both these factors was .61, supporting their discriminant validity.

Score reliability results (Table 4) echoed those from convergent validity: score reliability for OM was acceptable, while that for SM was below the acceptable threshold.

**Table 4.** Measurement Invariance Tests Across Sex for the MOBAK Model 2c (2 Residual Covariances)

Model	χ^2^(df)	CFI	RMSEA [90% CI]	ΔCFI	ΔRMSEA	Robust Δχ^2^ difference test (df), *p*-value
MI1. Configural	57.99 (38)	.964	.049 [.020, .074]	—	—	—
MI2.Threshold-only	57.99 (38)	.964	.049 [.020, .074]	0	0	—
MI3. Thresholds + Loadings	78.96 (44)	.936	.061 [.038, .082]	−.028	.012	19.31 (6), *p* = .004
MI4. Partial Thresholds + Loadings	65.28 (42)	.958	.051 [.024, .073]	−.006	.002	7.41 (4), *p* = .116

*Note.* Female *n* = 203; Male *n* = 233; CFI = Comparative Fit Index; RMSEA = Root Mean Square Error of Approximation; Δ values are relative to the less restricted model immediately above, except for MI4 where comparison is made against MI2.

Scaled indices are presented.

#### Measurement Invariance

To ensure psychometric quality and comparability of constructs, measurement invariance was tested across sex (Table 4) as this is a key grouping variable in children’s motor competence research and a primary source of expected differences (e.g., Barnett, Lai, et al., 2016).

Configural invariance (MI1) of the two-factor MOBAK model (including two residual covariances) across sex was supported, with overall acceptable approximate fit (*p* = .02, good CFI, RMSEA). As noted by Wu and Estabrook (2016), thresholds-only invariance (MI2) cannot be formally tested with three-category ordinal indicators, which was consistent with our finding of no differences between configural and thresholds-only models. When constraining both thresholds and loadings (MI3), model fit significantly worsened (LRT, ΔCFI, ΔRMSEA), indicating non-invariant loadings. Modification indices highlighted Running (MI = 10.65) and Throwing (MI = 12.49) as the primary sources. We selected Running as the first candidate for freeing its loading, given its recurrent role in driving misfit in previous models; doing so improved fit to an acceptable level, and subsequent release of Throwing yielded no further change in indices but provided a cleaner residual structure. Thus, partial metric invariance was established, permitting valid latent mean comparisons while acknowledging item-level non-invariance (see, e.g., Byrne et al., 1989). In this final solution, Throwing loaded more strongly on OM for girls (≈.48 vs. ≈.21 in boys), while Running loaded more strongly on SM for boys (≈.46 vs. ≈.33 in girls.) Also notably, factor correlations among girls remained extremely high (≈.98) across models, suggesting a one-factor structure may better capture their motor skill organization (see Discussion).

#### Know-Groups Validity

##### Sex-Related Effects on Basic Motor Competencies

Latent means were compared under the partial thresholds and loadings invariance model, fixing the female group as reference (Table 5).

**Table 5.** Latent Mean Estimates Using Partial Thresholds and Loadings Invariance Model

Latent factor	*β* female	*β* Male	SE	z	*p*	Std. Diff. (d) [95% CI]
Object Movement	—	1.25	0.20	6.30	<.001	0.87 [0.86, 1.63]
Self-Movement	—	−0.26	0.16	−1.60	.11	−0.29 [−0.57, 0.06]

*Note.* Female (*n* = 203) was used as the reference group, with male (*n* = 233) latent means estimated relative to this group under the partial thresholds + loadings invariance model (Throwing and Running loadings freed). β = unstandardised latent mean estimate; SE = standard error; z = Wald test statistic for group mean difference; *p* = two-tailed p-value; Std. Diff. (d) = standardised mean difference (Cohen’s d) with 95% confidence interval.

Males scored significantly higher on OM, as indicated both by statistical significance and a large effect size (Cohen, 1988). No significant sex difference emerged for SM (no statistical significance, low effect), suggesting broadly comparable performance across groups in this domain.

##### Age and Sex-Related Effects on Basic Motor Competencies

**Figure 3.**
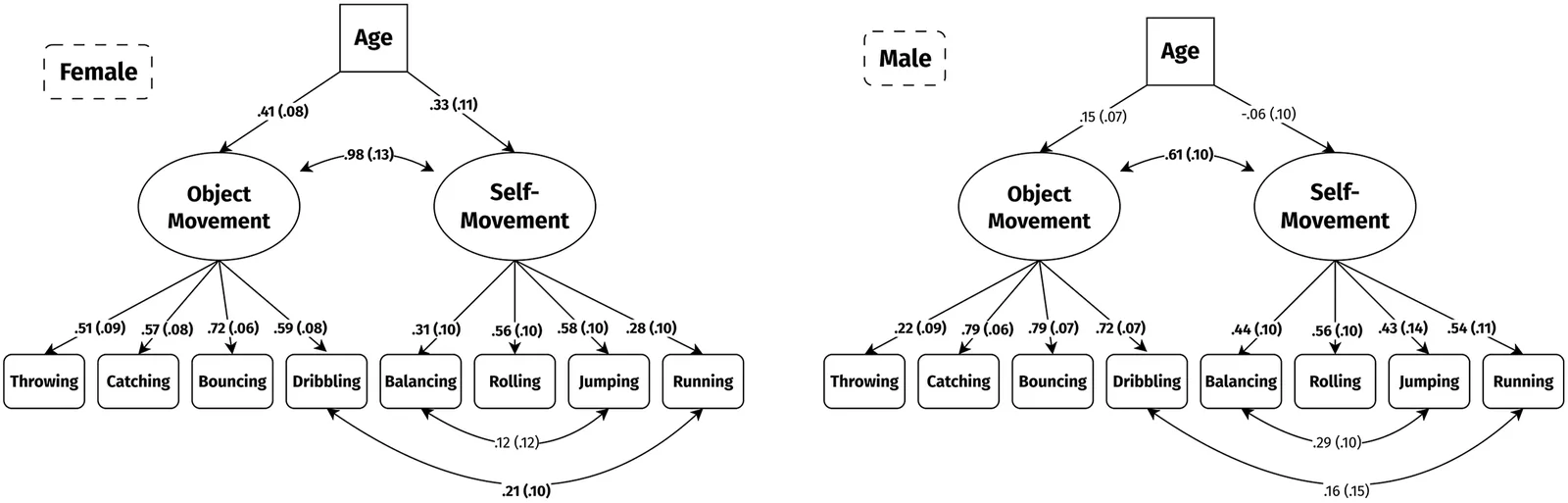
MIMIC-Type Multiple Group Partial Invariant Model (Sex) With Age as Covariate. Fully Standardized Solution (Please Note That Invariant Items Were Constrained in Their Unstandardized Loadings). Statistically Significant Values are Bolded

To explore age-related effects, a MIMIC model including age as an observed covariate of the partial invariant model 2c was fitted (see Figure 3). This resulted in a reasonable fitting model, albeit not passing the exact fit test (Scaled χ^2^ (50) = 83.202, *p* = .002), demonstrating borderline acceptable approximate fit according to all scaled indices (CFI = .94, RMSEA = .06 [.03, .08], SRMR = .06). Inspection of MI suggested a limited set of local strains, primarily among SM indicators (e.g., Balancing–Rolling MI = 7.76; Rolling–Running MI = 4.20) and between OM tasks (Throwing–Dribbling MI = 4.32; Bouncing–Dribbling MI = 3.22), along with suggested cross-loadings for Throwing and Balancing on the SM and OM factor, respectively (MIs = 6.35 and 3.32); all without substantive theoretical justification. Standardized residuals corroborated this pattern, suggesting modest item-level overlap—on top of a complex model— rather than substantive local misfit. The full residual matrix is provided in Supplemental Table 3 and Supplemental Table 4 for transparency.

In girls, age was positively and significantly associated with both OM and SM, indicating that these skills tend to improve moderately as they get older. For boys, however, age showed only a weak, non-statistically significant positive link with OM (p = .060) and no meaningful association with SM (*p* = .515), suggesting little to no improvement in these skills with age. These patterns point to sex as a potential moderator of the age–MOBAK relationship. To test this formally, we compared a model that allowed age slopes to differ by sex with one that constrained them to be equal. The free-slopes model fit the data significantly better than the constrained model (Robust χ^2^(2) difference = 7.72, *p* = .02), supporting the moderating role of sex.

## Discussion

### Construct Validity

#### Structural, Convergent, Discriminant Validity

Our results revealed that the unidimensional model (M1) did not fit the data well. In contrast, the two-correlated factors model (Model 2a) showed a better fit, though it still had some limitations. By iteratively addressing specific areas of local strain and respecifying the model, we found model 2c to be the best global fit to our data—a two-correlated factor solution with two residual covariances. Sensitivity analysis of models with cross-loadings and item removal demonstrated that doing so would benefit overall model fit, at the cost of content coverage and loss of compatibility of results across studies using this battery.

Overall discriminant validity was generally supported by the moderate correlation between the two factors in the final solution, and was similar in magnitude to those reported in other studies (Carcamo-Oyarzun & Herrman, 2020; Herrmann & Seelig, 2017; Šiška et al., 2024). Convergent validity assessed through factor loadings was good for the OM factor but less so for the SM factor. This may reflect the diversity of motor patterns assessed within SM. While Jumping involves a prolonged cyclical action on the spot, both Rolling and Balancing require acyclic movements executed over a defined linear course. The Running test differs further, as it combines forward running with sidesteps in a set pattern, requiring dynamic changes in direction and coordination across locomotor planes. Moreover, while Jumping demands synchronization with an external object (rope), Rolling, Balancing, and Running involve movement of the child in relation to static environmental structures (mat, bench, cones/pattern). While attempting to capture such distinct patterns under a single broad factor presents benefits for breadth of monitoring MC development, nuances in the underlying capacities elicited and ordinal scoring format used might present difficulties for model fit and reduce the variance explained by the latent factor, resulting in low score reliability for this factor —which have been noted in the battery’s website (MOBAK, 2025) and can be inferred from the loadings obtained across other validation studies (Carcamo-Oyarzun & Herrman, 2020; Herrmann & Seelig, 2017; Šiška et al., 2024). Further research efforts should look to (a) refine these tests by minimizing construct-irrelevant variation, while maintaining the intended ecological validity of the MOBAK 3-4 (Herrmann et al., 2015); (b) investigate alternative modelling frameworks based on Item Response Theory (e.g., 2-parameter or Graded Response models) which were designed to inherent deal with ordinal scoring formats (De Ayala, 2009); (c) consider revision of the scoring methodology to fully account for the number of successful attempts to potentially expand available variance and test score reliability.

#### Measurement Invariance Across Sex and Score Reliability

Our results indicate that the MOBAK 3–4 two-factor model is partially invariant across sex (MI4). This permits meaningful comparison of latent means while recognizing that Throwing and Running displayed variant loadings (the former stronger in girls, the latter in boys), reflecting sex-differential developmental patterns. Although novel for MOBAK, this pattern mirrors broader findings in motor-assessment psychometrics, where full sex invariance is uncommon (Aadland et al., 2022; Birklbauer et al., 2024).

Portuguese extracurricular participation profiles at this age might help explain the differential loadings. Girls’ ball-sport involvement is less football-centric and more dispersed across football, basketball, handball, and volleyball (Direção Geral de Estatísticas da Educação e Ciência [DGEEC] & Divisão de Estudos e de Gestão do Acesso a Dados para Investigação [DEGADI], 2020), yielding a more balanced OM skill set; consequently, Throwing co-varies more with Catching, Bouncing, and Dribbling, elevating its loading on OM. By contrast, boys’ concentrated football participation (with basketball a distant second) offers little direct transfer to overarm throwing, reducing Throwing’s shared variance with the other OM indicators even though OM remains well defined by Catching/Bouncing/Dribbling. For Running, the lower loading in girls reflects two converging features: (i) overall, girls are more likely to participate in activities that tighten covariance among Balancing/Rolling/Jumping (e.g., dance/gymnastics) rather than with the MOBAK Running format which centers on directional change; and (ii) the subgroup of girls who do engage in ball sports likely get to develop change-of-direction ability, on top of the more balanced OM skillset mentioned above, siphoning variance away from SM. This interpretation is consistent with evidence that sex-differentiated engagement profiles shape object-control and locomotion proficiency and its correlates (e.g., Barnett, Lai, et al., 2016). Further work should seek to replicate these findings across different sport participation ecologies.

Factor correlations in model MI4 differed markedly by sex: near unity among girls (r ≈ .99) versus moderate among boys (r ≈ .55). This raises concerns about discriminant validity in girls, suggesting that MOBAK may capture a more generalized motor competence factor in this group rather than two distinct domains. These sex-specific differences indicate a structurally weaker two-factor solution for girls, even though configural invariance was met. The inflated correlation likely reflects reduced distinctiveness between factors in girls, driven by the proposed mechanisms above, alongside a set of heterogeneous motor demands within SM that weaken its internal cohesion.

Our reliability and validity results provide no support for a single total MOBAK score, given the poor fit and low internal consistency of a unidimensional model. OM demonstrated acceptable convergent validity and reliability, whereas SM was weaker, precluding robust interpretation as a stand-alone scale, especially for girls. For high-stakes or research applications, Structural Equation Modelling-based approaches remain recommended to directly account for measurement error and the observed structural differences (Kline, 2023). Alternatively, regression-weighted scores are an apt middle ground solution, as they respect differential item weights and residual structure, without requiring direct use of specialized software (DiStefano et al., 2009; Grice, 2001)—formulas to calculate these are provided in Supplemental File 2, and an Excel-based scoring tool is in development to further support practitioners. For applied settings, summed subscores may offer teachers a rapid way to monitor pupils’ motor competence, but not without caveats derived both from their general psychometric limitations (McNeish & Wolf, 2020) and current results: in our data, sum–factor score correlations were high, particularly for boys (ρ ≈ .94 and .90), supporting sums as workable proxies for their latent ability. For girls, however, we found a weaker correspondence for SM (ρ ≈ .76; OM ρ ≈ .94), mirroring the near-unity OM–SM correlation, indicating little distinct variance, and highlighting that using regression-based scores might produce a more nuanced and accurate representation of their latent ability. Full results for these correlations are available in Supplemental Table 5.

Taken together, our findings indicate that while the MOBAK’s two-factor structure is psychometrically defensible and preferable overall, its functioning differs across sexes. For applied practice, both subscores should be reported and interpreted together—preferably using the regression-weighted scores—but in girls they may best be understood as overlapping reflections of general competence. Future work should further examine score reliability in SM, and test whether a unidimensional or bifactor model may be suitable for girls in certain contexts, as has been reported in other motor competence batteries (e.g., TGMD-3: Salami et al., 2022; Garn & Webster, 2021).

#### Known-Groups Validity: Sex and Age

The present findings from our latent means comparison and MIMIC model provide overall support for the known-groups validity of MOBAK 3-4 in Portuguese children, revealing sex-specific developmental patterns in BMC. Consistent with established research, boys outperformed girls in manipulative (OM) skills (Barnett et al., 2010, Barnett, Lai, et al., 2016; Bolger et al., 2021; Carcamo-Oyarzun & Herrman, 2020; Quitério et al., 2018; Scheuer et al., 2017, Scheuer, Bund, & Herrmann, 2019; Šiška et al., 2024; Strotmeyer et al., 2020; Wälti et al., 2022), while evidence for sex differences in locomotor and stability (SM) skills remains inconsistent across studies.

Crucially, age-related improvements varied significantly by sex. In girls, age was positively and significantly associated with both OM and SM, indicating moderate skill improvement over time. For boys, however, age showed only a weak positive link with OM and no meaningful association with SM, suggesting minimal age-related development. These patterns robustly demonstrate that sex moderates the age–MOBAK relationship, with girls showing consistent developmental gains while boys show minimal improvement, consistent with evidence that fundamental motor skills development might plateau during childhood (Valentini et al., 2016) and that such moderation might happen in particular age ranges; a fact that warrants further investigation. This divergence also reflects that motor development is age-related rather than age-dependent, with environmental factors playing crucial roles (SHAPE America, 2025). Since maturation shows weak associations with MC in prepubertal children given similar body characteristics across sexes (Malina et al., 2004), activity preferences become influential, as previously explored. These findings suggest targeted interventions should provide structured practice opportunities, particularly in manipulative skills for girls, while recognizing that children of the same age and sex may occupy different developmental stages, necessitating differentiated instruction.

Although our partially invariant model achieved acceptable fit, residual diagnostics revealed possible age-related invariance in Running, Jumping, and Throwing items, suggesting developmental progressions may not be fully captured by the latent structure. Future research should examine these age-related residuals and investigate the role of other relevant covariates like Body Mass Index in MC developmental patterns (Wälti et al., 2025).

### Strengths and Limitations of This Study

This study offers several strengths that enhance its robustness. The inclusion of both intra- and inter-observer reliability checks adds methodological rigor and provides strong evidence against observer bias. We also conducted comprehensive construct validity analyses, including CFA, and, to our knowledge, the first formal test of measurement invariance across sex, alongside an evaluation of known-group validity using a large, representative sample drawn from multiple schools in the Cascais municipality, which allowed us to reveal meaningful sex moderation effects.

However, limitations must be acknowledged. Our selected residual covariances and item-level non-invariance added to the original MOBAK 3-4 model, despite theory-informed, carry the potential risk of model overfitting, and the findings should be interpreted with caution until replicated in independent and cross-cultural samples to ensure generalizability and to strengthen the international validity claims of MOBAK 3–4. Also, it is plausible that class and school clustering effects are present, which we did not account for; future work should consider this using, e.g., multilevel models.

## Conclusions

Supported by trained test administrators and a large representative sample, this study provides robust evidence for the structural, convergent, and discriminant validity, score reliability, and known-groups validity of the MOBAK 3–4 as a tool to assess BMC in Portuguese children aged 8–10 years, A two-factor model (with OM and SM) was confirmed, though partial measurement invariance across sex emerged, with Throwing and Running showing sex-differential loadings, and near-unidimensionality in girls. These findings suggest that the MOBAK functions differently across sexes but still permits valid latent mean comparisons and is able to detect meaningful age- and sex-related differences in motor competence.

Score reliability analyses indicated that summed scores are acceptable for rapid monitoring—particularly in boys—but are less robust for girls due to reduced discriminant validity. Regression-weighted scores provide a stronger applied alternative, while SEM-derived scores remain preferable for research. Importantly, a total MOBAK score is not supported and isolated subscales should be interpreted with caution, especially for SM, and when comparing across sexes.

In practice, teachers can use subscores to monitor progress in PE, but researchers and policymakers should account for sex-specific measurement properties when interpreting results. By refining scoring practices and integrating MOBAK with broader dimensions of physical literacy, this tool can meaningfully contribute to the monitoring and promotion of lifelong physical activity.

## Supplemental Material

Supplemental Material - MOBAK 3–4: Construct Validity and Score Reliability in an 8–10-Year-Old Portuguese Sample Within the Cascais Municipality Supplemental Material for MOBAK 3–4: Construct Validity and Score Reliability in an 8–10-Year-Old Portuguese Sample Within the Cascais Municipality by João Mota, Afonso Meira, João Martins, Marcos Onofre and Maria João Martins in Perceptual and Motor Skills.

Supplemental Material - MOBAK 3–4: Construct Validity and Score Reliability in an 8–10-Year-Old Portuguese Sample Within the Cascais Municipality Supplemental Material for MOBAK 3–4: Construct Validity and Score Reliability in an 8–10-Year-Old Portuguese Sample Within the Cascais Municipality by João Mota, Afonso Meira, João Martins, Marcos Onofre and Maria João Martins in Perceptual and Motor Skills.
