# Supplementary material for: MOBAK 3–4: Construct Validity and Score Reliability in an 8–10-Year-Old Portuguese Sample Within the Cascais Municipality
Source: Percept Mot Skills. 2025 Dec 18;133(5):1283–304. doi: 10.1177/00315125251401274 (PMC13569896; doi:10.1177/00315125251401274)
Supplement: Supplementary file 1 [file sj-pdf-1-pms-10.1177_00315125251401274.pdf]

Supplemental File 1

Supplemental Table 1. Item Score Distribution (N=436)

	Female (N = 203)	Male (N = 233)	Female (N = 203)	Male (N = 233)	Female (N = 203)	Male (N = 233)	Female (N = 203)	Male (N = 233)
	**Throwing**		**Catching**		**Bouncing**		**Dribbling**	
0	119 (59%)	109 (47%)	95 (47%)	56 (24%)	88 (43%)	42 (18%)	65 (32%)	37 (16%)
1	70 (34%)	103 (44%)	68 (33%)	59 (25%)	45 (22%)	53 (23%)	70 (34%)	56 (24%)
2	14 (6.9%)	21 (9.0%)	40 (20%)	118 (51%)	70 (34%)	138 (59%)	68 (33%)	140 (60%)
	**Balancing**		**Rolling**		**Jumping**		**Running**	
0	59 (29%)	82 (35%)	85 (42%)	95 (41%)	117 (58%)	185 (79%)	19 (9.4%)	25 (11%)
1	62 (31%)	65 (28%)	25 (12%)	40 (17%)	52 (26%)	30 (13%)	48 (24%)	39 (17%)
2	82 (40%)	86 (37%)	93 (46%)	98 (42%)	34 (17%)	18 (7.7%)	136 (67%)	169 (73%)

Note. n (%)

Supplemental Table 2. Correlation residuals matrix for Model 2c

	Throwing	Catching	Bouncing	Dribbling	Balancing	Rolling	Jumping
**Throwing**	—						
**Catching**	.055	—					
**Bouncing**	.011	-.014	—				
**Dribbling**	-.093	-.012	.029	—			
**Balancing**	.040	-.015	-.037	–.011	—		
**Rolling**	-.056	.019	-.056	.004	.057	—	
**Jumping**	.059	**-.104**	.044	.030	.000	-.049	—
**Running**	.034	**.121**	.006	.000	-.047	-.032	-.112

Note. Values simultaneously higher than |.10| and presenting a z-value higher than |1.96| are bolded.

Supplemental Table 3. Correlation residuals matrix for MIMIC Model (Female)

	Throwing	Catching	Bouncing	Dribbling	Balancing	Rolling	Jumping
**Throwing**	—						
**Catching**	.102	—					
**Bouncing**	-.034	.027	—				
**Dribbling**	**-.165**	-.119	.097	—			
**Balancing**	.021	-.031	.028	.037	—		
**Rolling**	-.024	.110	-.092	-.008	-.032	—	
**Jumping**	-.070	-.103	.031	.088	.000	.005	—
**Running**	-.029	.061	.057	.000	-.070	.146	-.092

Note: Values simultaneously higher than |.10| and presenting an approximate z-value higher than |1.96| are bolded.

Supplemental Table 4. Correlation residuals matrix for MIMIC Model (Male)

	Throwing	Catching	Bouncing	Dribbling	Balancing	Rolling	Jumping
**Throwing**	—						
**Catching**	.036	—					
**Bouncing**	.048	-.025	—				
**Dribbling**	-.081	.025	.007	—			
**Balancing**	.024	.011	-.100	-.072	—		
**Rolling**	.152	.028	-.023	.009	**.145**	—	
**Jumping**	.076	.022	.066	-.029	.000	.073	—
**Running**	.072	.122	.074	.000	-.049	**-.136**	.123

Note: Values simultaneously higher than |.10| and presenting an approximate z-value higher than |1.96| are bolded.
