# Supplementary material for: MOBAK 3–4: Construct Validity and Score Reliability in an 8–10-Year-Old Portuguese Sample Within the Cascais Municipality
Source: Percept Mot Skills. 2025 Dec 18;133(5):1283–304. doi: 10.1177/00315125251401274 (PMC13569896; doi:10.1177/00315125251401274)
Supplement: Supplementary file 2 [file sj-pdf-2-pms-10.1177_00315125251401274.pdf]

Supplemental File 2

Regression factor scores were calculated using the Thomson method, which derives item weights from the complete confirmatory factor analysis results. This approach incorporates factor loadings (both constrained and freed parameters from our partial metric invariance model), factor variances and correlations, and residual variances into a unified weighting system. The method inverts the model-implied covariance matrix to obtain optimal linear predictors rather than relying solely on factor loadings. Additionally, because items were measured on ordinal scales, Thomson weights were adjusted using threshold linearization to account for non-equal intervals in the 0-1-2 rating scale. Finally, weights were rescaled so that each factor's maximum possible score remained 8 (matching the original sum score range of 0-8), enabling practical field scoring while preserving the psychometric properties of the full measurement model (Grice, 2001; DiStefano et al., 2009).

Calculating regression-based factor scores:

Female scoring formulas:

- **Object Movement** = Throwing*0.488 + Catching*0.600 + Bouncing*0.799 + Dribbling*0.658 + Balancing*0.252 + Rolling*0.404 + Jumping*0.548 + Running*0.251
- **Self-Movement** = Balancing*0.266 + Rolling*0.425 + Jumping*0.577 + Running*0.264 + Throwing*0.473 + Catching*0.581 + Bouncing*0.775 + Dribbling*0.638

Male scoring formulas:

- **Object Movement** = Throwing*0.047 + Catching*0.521 + Bouncing*2.597 + Dribbling*0.684 + Balancing*0.019 + Rolling*0.038 + Jumping*0.060 + Running*0.034
- **Self-Movement =** Balancing*0.438 + Rolling*0.868 + Jumping*1.378 + Running*0.769 + Throwing*0.007 + Catching*0.074 + Bouncing*0.369 + Dribbling*0.097

Supplemental Table 5. Spearman correlations of Regression-Based scores derived from partially invariant model MI4 and Summed Scores

**Factor**	**Female ρ [95% CI]**	**Male ρ [95% CI]**	**Difference [95% CI], p-value**
Object Movement	.94 [.92, .95]	.90 [.86, .92]	.04 [.01, .08], p = .021
Self-Movement	.76 [.70, .82]	.94 [.92, .96]	-.18 [-.25, -.12], p <.001

Note. Confidence intervals obtained through bias-corrected bootstrapping with 2000 replications.

References

DiStefano, C., Zhu, M., & Mîndrilă, D. (2009). Understanding and using factor scores: Considerations for the applied researcher. *Practical Assessment, Research & Evaluation, 14*(20), 1–11.

Grice, J. W. (2001). Computing and evaluating factor scores. *Psychological Methods, 6*(4), 430–450. https://doi.org/10.1037/1082-989X.6.4.430
